# Soluble T-cell receptor design influences functional yield in an *E*. *coli* chaperone-assisted expression system

**DOI:** 10.1371/journal.pone.0195868

**Published:** 2018-04-12

**Authors:** Kristin Støen Gunnarsen, Lene Støkken Høydahl, Ralf Stefan Neumann, Kaare Bjerregaard-Andersen, Nicolay Rustad Nilssen, Ludvig Magne Sollid, Inger Sandlie, Geir Åge Løset

**Affiliations:** 1 Centre for Immune Regulation, University of Oslo and Oslo University Hospital-Rikshospitalet, Oslo, Norway; 2 Department of Immunology, University of Oslo and Oslo University Hospital-Rikshospitalet, Oslo, Norway; 3 Department of Biosciences, University of Oslo, Oslo, Norway; 4 Department of Chemistry, University of Oslo, Oslo, Norway; 5 KG Jebsen Coeliac Disease Research Centre and Department of Immunology, University of Oslo, Oslo, Norway; 6 Nextera AS, Oslo, Norway; J. Heyrovsky Institute of Physical Chemistry, CZECH REPUBLIC

## Abstract

There is a quest for production of soluble protein of high quality for the study of T-cell receptors (TCRs), but expression often results in low yields of functional molecules. In this study, we used an *E*. *coli* chaperone-assisted periplasmic production system and compared expression of 4 different soluble TCR formats: single-chain TCR (scTCR), two different disulfide-linked TCR (dsTCR) formats, and chimeric Fab (cFab). A stabilized version of scTCR was also included. Additionally, we evaluated the influence of host (XL1-Blue or RosettaBlue^TM^) and the effect of IPTG induction on expression profiles. A celiac disease patient-derived TCR with specificity for gluten was used, and we achieved detectable expression for all formats and variants. We found that expression in RosettaBlue^TM^ without IPTG induction resulted in the highest periplasmic yields. Moreover, after large-scale expression and protein purification, only the scTCR format was obtained in high yields. Importantly, stability engineering of the scTCR was a prerequisite for obtaining reliable biophysical characterization of the TCR-pMHC interaction. The scTCR format is readily compatible with high-throughput screening approaches that may enable both development of reagents allowing for defined peptide MHC (pMHC) characterization and discovery of potential novel therapeutic leads.

## Introduction

The T-cell receptor (TCR) plays a central role in adaptive immunity by mediating recognition of peptides presented by the major histocompatibility complex (MHC) on the surface of antigen presenting cells. Studies of the interaction between individual TCRs and their specific peptide MHC (pMHC) complexes continue to give insights into the biological functions of T cells, as well as information necessary for the design and safeguarding of TCR-based therapeutics [1–3]. Production of soluble TCRs of high quality and in high yields is therefore needed for biophysical characterization of TCR interactions. Furthermore, soluble TCRs are useful as detection reagents when studying antigen presentation [4–6]. Compared to the structurally similar antibody molecules, TCRs are generally less stable when expressed as soluble molecules, and problems such as low expression yields, aggregation, misfolding and inefficient chain pairing are often encountered [7–9].

Different soluble TCR formats have been constructed, such as single-chain TCR (scTCR), where the two variable domains are connected through a flexible linker, fusion of the TCR α- and β-chains to other proteins, such as leucine zippers, the human constant antibody kappa domain or the TCR constant β domain, and by introduction of a non-native disulfide bond between the TCR constant domains to generate disulfide-linked TCRs (dsTCRs) [10–15]. Production of dsTCRs has been the most successful strategy, but the molecules have only been expressed in the *E*. *coli* cytosol, and thus at reducing conditions not compatible with efficient disulfide bond formation and chain pairing. An attempt to overcome this by use of a modified *E*. *coli* strain had limited success, and one therefore relies on refolding of cytosolic inclusion bodies for production, which is time-consuming and not compatible with high-throughput approaches [13, 16].

High throughput approaches are desirable whenever engineering is carried out to increase the stability, affinity or production yields by phage or yeast display. Therefore, an attractive alternative is protein targeting to the oxidizing periplasm of *E*. *coli* with simultaneous co-expression of chaperones such as FkpA, and which is compatible with large-scale library screening after display selections [17]. Such periplasmic targeting has resulted in greatly increased functional expression yields of several scTCRs [17–20]. However, whether or not other TCR formats can be produced by periplasmic targeting, has not been investigated.

In the current study, we wanted to study how different TCR formats were expressed after targeting to the periplasmic space with FkpA folding assistance. We aimed to produce high quality, soluble TCR for biophysical studies of the TCR-pMHC interaction. To this end, we constructed soluble TCRs of 4 different formats, namely the two previously described formats, scTCR and dsTCR, as well as two novel formats, which we denote cFab (chimeric Fab) and ct-dsTCR (c-terminal dsTCR). We also included a scTCR variant containing stabilizing mutations. We compared the expression yield, stability and purity of the variants, as wells as the importance of expression host (XL1-Blue or RosettaBlue^TM^) and expression conditions with or without IPTG induction. We observed expression of all formats and variants, and identified the most successful combination of format and conditions. The stabilized scTCR expressed in RosettaBlue^TM^ without IPTG induction, enabled production of sufficient quantities of soluble TCR for pMHC interaction studies.

## Results

### TCR construct design

**Fig 1:**
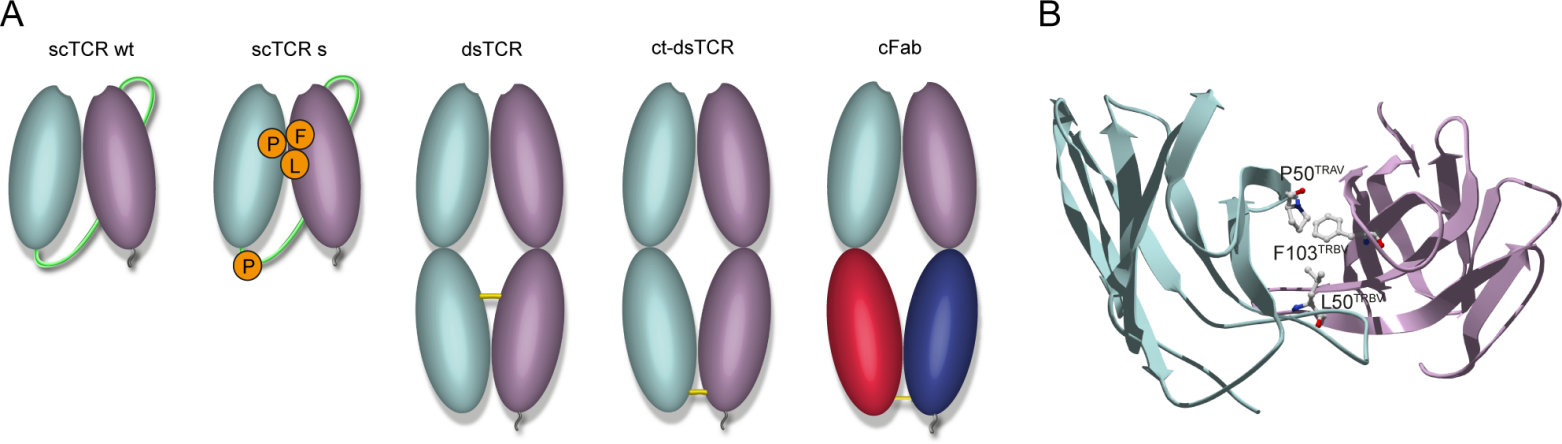
Overview of the TCR formats and triad of amino acids that form a stabilized interphase. (A) Schematic presentation of the TCR formats. From left to right; scTCR wt, scTCR s, dsTCR, ct-dsTCR and cFab. TCR α- and β-chains are colored pale green and purple, respectively, and C_H_1 and C_κ_ of the cFab are colored red and blue, respectively. The Vα/Vβ domains of the scTCRs are connected via a peptide linker as shown in green. Disulfide bonds are illustrated in yellow and the c-Myc and His6 -tags are shown in grey. (B) Top-down view of the amino acids forming the Vα/Vβ interphase triad, P50^TRAV^, L50^TRBV^ and F103^TRBV^. The crystal structure of the HLA-DQ2.5-DQ2.5-glia-α2-specific TCR S16 was used for modelling the triad (PDB ID 4OZH) [25].

In order to obtain soluble monomeric TCR of high quality and yield using expression conditions that are compatible with high throughput production of variants, we compared periplasmic *E*. *coli* expression of different molecular formats with co-expression of the chaperone FkpA [17, 20]. Using the celiac disease-associated HLA-DQ2.5:DQ2.5-glia-α1a-restricted TCR380 as template [21], we constructed 5 variants as schematically illustrated in Fig 1A. The scTCR construct was generated by connecting the variable domains of the TCR with a flexible linker in a Vα-linker-Vβ orientation which was found to be the preferred orientation in a previous study [20]. We generated two variants of this format, wt380 and a stabilized version, s380, containing mutations predicted or shown to impact on stability (Fig 1A and detailed in S1A and S1B Fig) [17, 22]. The dsTCR was generated by introducing a disulfide bridge in the invariant region of the constant domains of the TCR [13], while the ct-dsTCR was engineered to contain the disulfide bridge-containing region from the antibody Fab fragment (Fig 1A and detailed in S1C and S1D Fig). Finally, the cFab format, a TCR/antibody chimeric Fab (cFab) fragment, was constructed by fusing Vα from the TCR to C_H_1 and the Vβ to C_K_ (Fig 1A and detailed in S1E Fig) [23, 24].

### Influence of host strain and expression condition on soluble TCR expression

**Fig 2:**
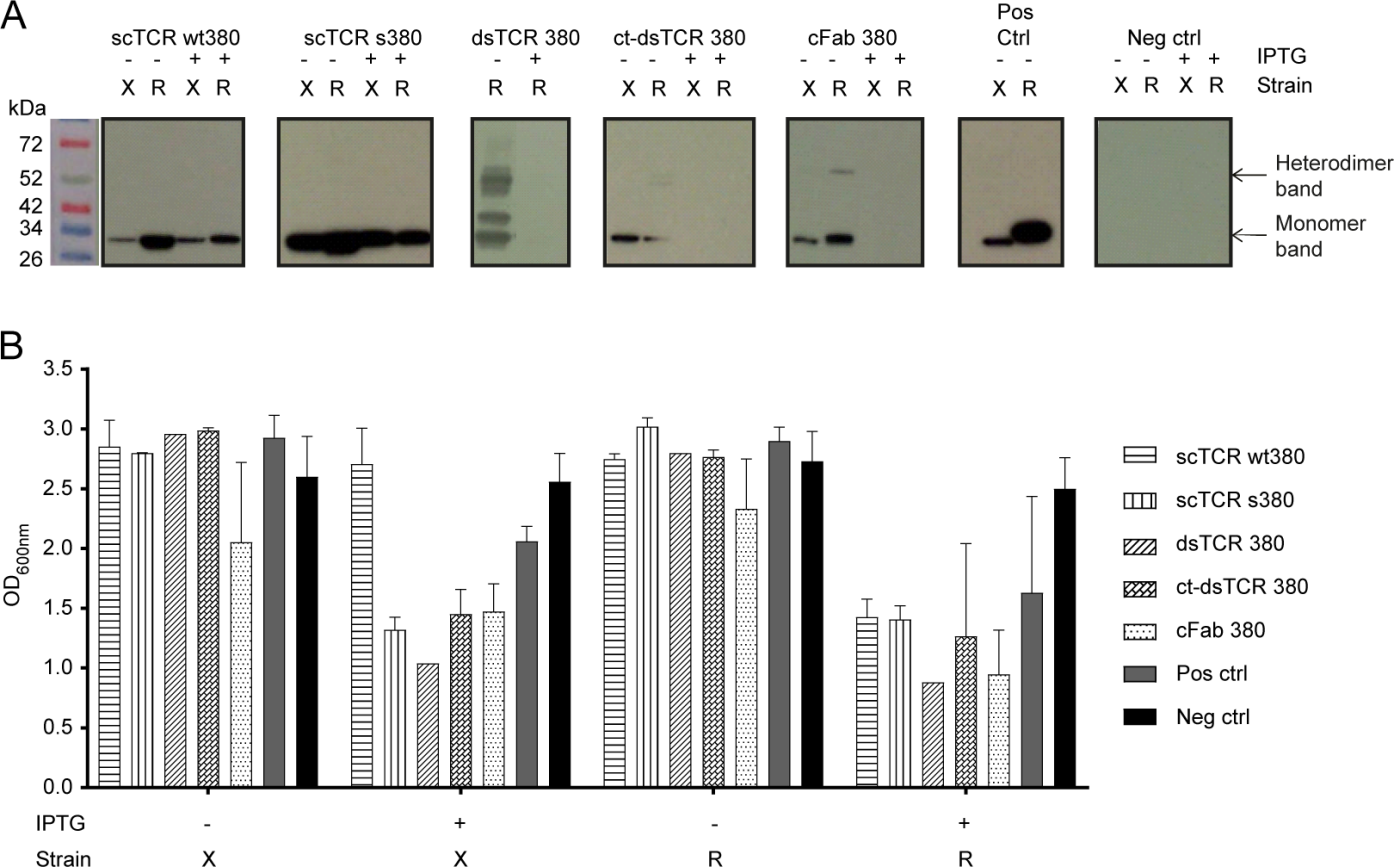
Effect of host strain and induction conditions on periplasmic TCR expression. (A) Representative western blots showing the expression profile of the TCR formats in either *E*. *coli* XL1-Blue (X) or RosettaBlue^TM^ (R) where IPTG induction of protein expression either was omitted (-) or included (+). Normalized expression cultures were fractionated before the presence of TCR in the periplasmic fractions was analyzed by western blot detection using an anti-His-HRP antibody (n = 3). (B) The bacterial growth characteristics were monitored by measuring the OD_600nm_ of *E*. *coli* XL1-blue (X) and RosettaBlue^TM^ (R) bacterial cultures after overnight protein expression without (-) or with (+) IPTG induction at 30°C. *n* = 3 for all constructs except for controls (*n* = 4) and the dsTCR constructs (*n* = 1). Standard deviations are indicated where possible. (A, B) Cells expressing scFv anti-phOx were used as positive control and untransformed cells as negative control.

We performed an initial small-scale *E*. *coli* periplasmic expression of the 5 different variants. Two different *E*. *coli* host strains were compared, XL1-Blue and RosettaBlue^TM^. XL1-Blue allows for efficient protein production and is a phage display compatible strain [20, 26–28]. RosettaBlue^TM^ is suited for protein production; in particular expression of eukaryotic proteins due to the expression of tRNAs for codons rarely used in *E*. *coli*. The variable region genes of TCR380 studied herein contain 16 such rare codons. Furthermore, the effect of including or omitting IPTG induction of protein expression was assessed. Overall, RosettaBlue^TM^ expression without IPTG induction resulted in the highest periplasmic expression yields (Fig 2A, S2 and S3 Figs). Under these expression conditions, heterodimer formation of dsTCR, cFab, and ct-dsTCR was detected. However, the ct-dsTCR band was very weak (Fig 2A and S3 Fig), and in some cases not even visible on the gel (S2 Fig). Thus, the ct-dsTCR was excluded from further analysis due to low yield of heterodimeric protein.

In general, IPTG induction reduced the expression levels, and for cFab, dsTCR and ct-dsTCR the expression was completely abrogated (Fig 2A). Expression in XL1-Blue was lower than in RosettaBlue^TM^, both in the presence and absence of induction (Fig 2A). Moreover, IPTG induction resulted in growth retardation in both XL1-Blue and RosettaBlue^TM^ (Fig 2B). Thus, after initial characterization of expression characteristics, both scTCR variants were readily expressed, while formation of the heterodimeric TCR formats resulted in less detectable protein.

### Effect of TCR format on expression characteristics

**Fig 3:**
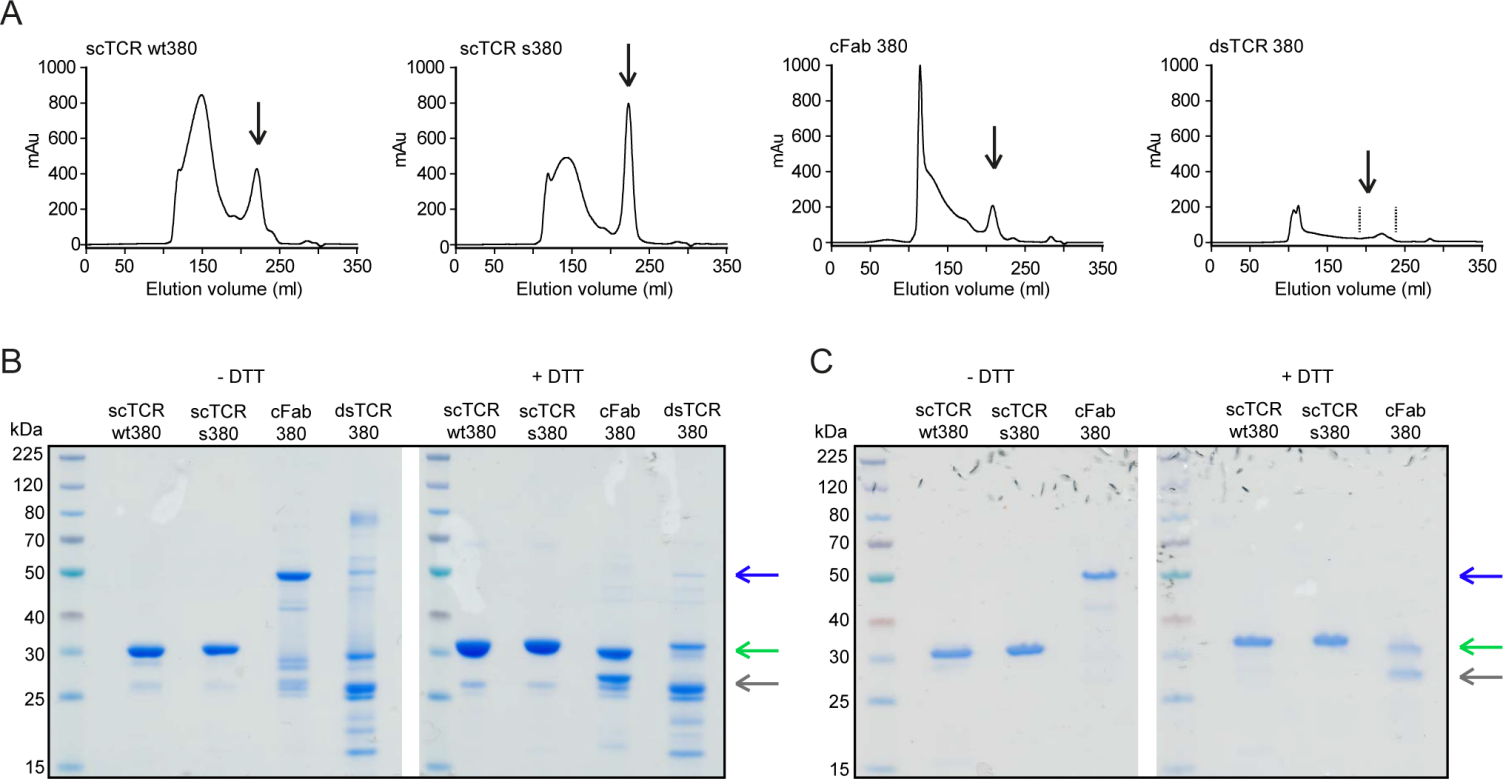
Purification profile and monomeric integrity of the expressed recombinant TCR formats. (A) Representative Superdex 200 SEC profiles of IMAC purified scTCR wt380, scTCR s380, cFab 380 and dsTCR 380 as indicated (*n* = 2–9). Elution volumes corresponding to the expected size of monomeric scTCRs and heterodimeric cFab 380 and dsTCR 380 are indicated by arrows. Notably, for the dsTCR we pooled several fractions as indicated by the dotted lines due to the lack of a clear peak indicating the elution volume of the dsTCR. (B) Representative non-reducing and reducing SDS-PAGE of SEC purified monomeric TCR fractions (*n* = 2). (C) Non-reducing and reducing SDS-PAGE of Resource Q/Superdex 75 purified monomeric and heterodimeric TCR fractions (*n* = 2). (B, C) Bands at about 32 kDa for the scTCRs, 55 kDa and 26/29 kDa for the cFab and 57 kDa and 25/32 kDa the dsTCR depending on non-reducing or reducing conditions are indicated with arrows (blue arrows for heterodimeric cFab and dsTCR, green arrows for scTCR and reduced β-chains and grey arrow for reduced α-chains.

To compare the 4 remaining variants with respect to production yield and purity, we purified His-tagged molecules by IMAC, followed by size exclusion chromatography (SEC). The final monomeric yields varied extensively, giving the hierarchy scTCR s380 (1.15 mg/L), scTCR wt380 (0.76 mg/L), cFab 380, (0.35 mg/L), and dsTCR 380 (0.14 mg/L) (Fig 3A and Table 1).

**Table 1:** Protein yields after each purification step.

TCR variant	Yield after IMAC	Yield after SEC	Yield after IEC
scTCR wt380	4.34 ± 2.04	0.76 ± 0.18	0.26 ± 0.23
scTCR s380	5.69 ± 1.92	1.15 ± 0.38	0.66 ± 0.12
cFab 380	3.19 ± 1.54	0.35 ± 0.04	0.03^a^
dsTCR 380	1.87 ± 0.90	0.14^a^	NA

Yields are shown as mg/L expression culture. The values are given as mean ± SD from 2–5 independent experiments. NA, not available.

^a^Due to low yields these purification steps were only repeated once.

We then analyzed the samples on SDS-PAGE (Fig 3B). Both wt and in particular s380 scTCR contained mostly monomeric protein of expected size. For the cFab, the dominant fraction was the covalent heterodimer. In contrast, the dsTCR was dominated by single chains and only a very weak band of such heterodimeric protein was detected, excluding this format from further analysis.

The remaining 3 variants were subjected to a final purification step by either ion exchange, or a new SEC resulting in highly pure samples. This came at the cost of loss of protein from all samples and in particular the cFab (Fig 3C and Table 1). Thus, after expression and purification the dsTCR and cFab variants were excluded.

### Effect of TCR format on protein stability

**Fig 4:**
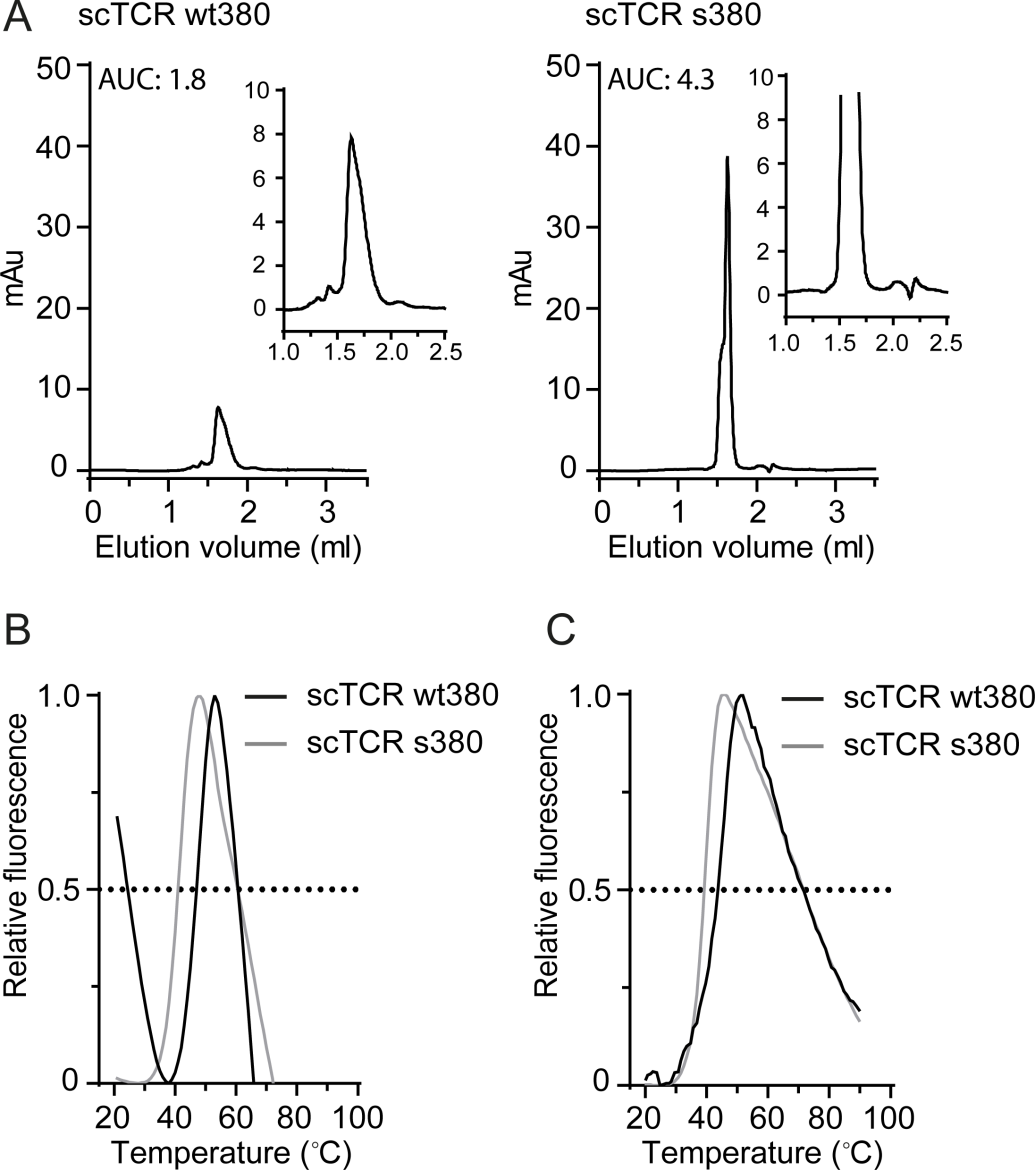
Stability assessment of the scTCRs. (A) Analytical SEC of scTCR wt380 and scTCR s380 to assess the integrity of the preparations after a freeze-thaw cycle (*n* = 2). Details of aggregates and monomeric peaks are shown in the magnified inset chromatograms. (B) DSF or (C) fluorescence spectroscopy measurements of scTCRs wt380 and s380 to calculate the melting temperature (T_m_) of the variants. The T_m_ values are summarized in Table 2 (*n* = 9–10 for DSF measurements and *n* = 2 for fluorescence spectroscopy measurements).

To investigate aggregation propensity and melting temperatures of scTCR wt380 and s380, we analyzed the isolated monomeric fractions by analytical SEC and differential scanning fluorimetry (DSF) after a freeze-thaw cycle. Despite application of equal amounts of protein of normalized concentration in the analytical SEC, the scTCR wt380 variant showed a reduced area under the curve (AUC) value compared to s380, indicating that a fraction of wt380 either precipitated in the column filter or nonspecifically adsorbed to or interacted with the matrix (Fig 4A). Thus, analytical SEC revealed that scTCR wt380, but not s380, showed signs of aggregation translating to a reduced amount of monomer. The melting curves of the scTCRs were analyzed by DSF and revealed average T_m_ values of the scTCR wt380 and s380 of 48.7°C and 43.3°C, respectively (Fig 4B and Table 2). Despite the fact that scTCR wt380 has the highest T_m_ value, the high initial fluorescence indicates that the wt380, and not s380, contained a fraction of partially unfolded proteins (Fig 4B).

**Table 2:** TCR stability assessment by DSF and fluorescence spectroscopy.

TCR variant	T_m_ (°C) (DSF)	T_m_ (°C)^a^
scTCR wt380	48.7 ± 1.4^b^	43.2 ± 1.5^c^
scTCR s380	43.3 ± 1.0^b^	39.2 ± 0.3^c^

^a^T_m_ from fluorescence spectroscopy

^b^The values are mean ± SD of 9–10 independent experiments

^c^The values are mean ± SD of 2 independent experiments.

To validate the DSF data, we determined the melting curves of the proteins based on intrinsic tryptophan fluorescence. The spectra revealed a single transition with a T_m_ of 43.2°C and 39.2°C for the scTCR wt380 and s380, respectively (Fig 4C and Table 2). Thus, both methods show the mutant form to be less thermostable than the wt with a 4°C lower T_m_. However, all data taken together points to aggregation of partially unfolded scTCR wt380, while the scTCR s380 remains in a uniform monomeric state.

### Functional binding and affinity measurements of the soluble TCRs

**Fig 5:**
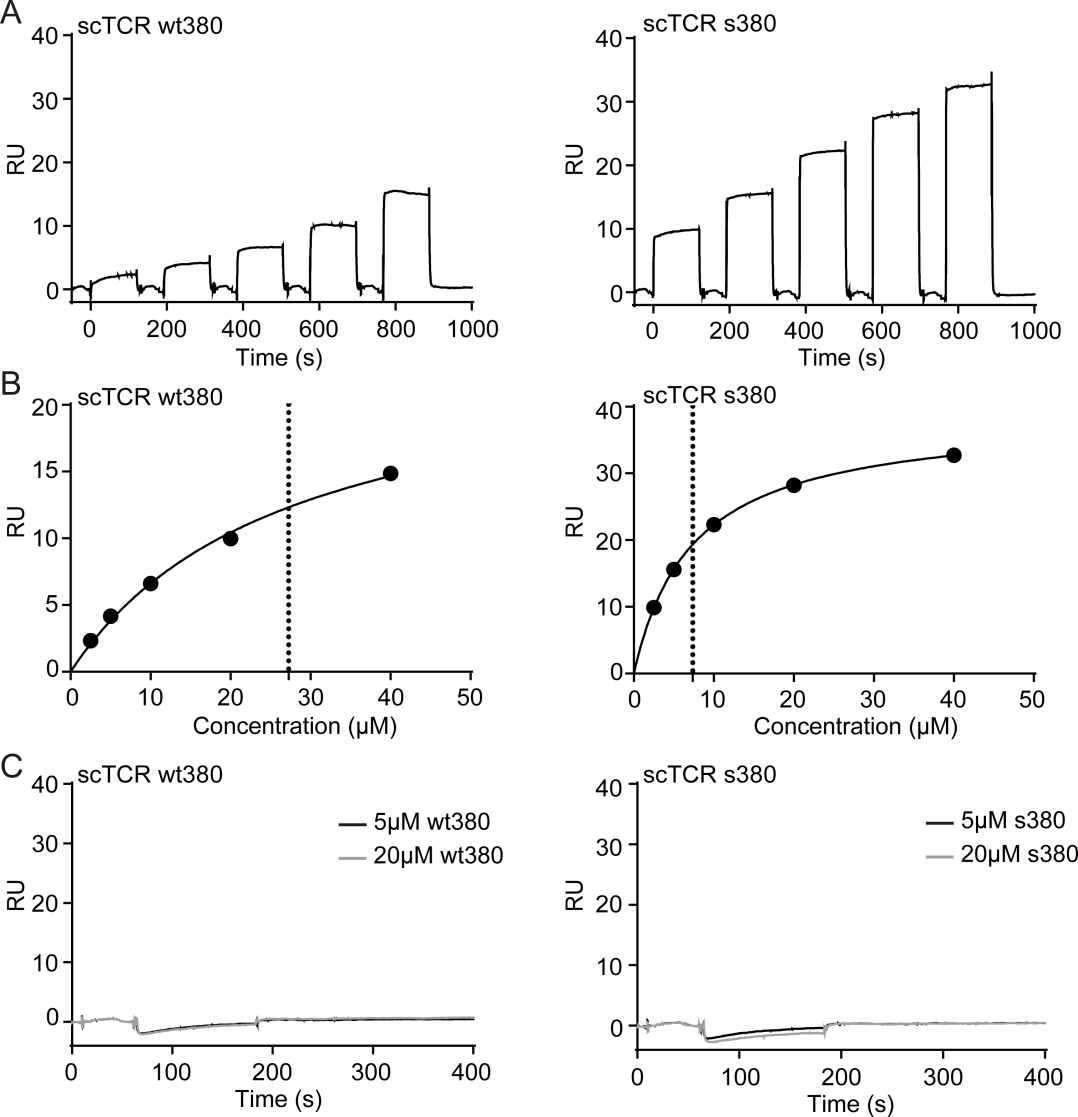
SPR binding characteristics of the scTCRs. (A) Biotinylated pMHC was captured on neutravidin-coated sensor chips followed by injection of a 2-fold dilution series from 40 **μ**M of scTCR. Representative sensograms of scTCR wt380 and scTCR s380 binding to HLA-DQ2.5:DQ2.5-glia-a1a (*n* = 1–3). (B) The equilibrium response was plotted against concentration to derive the equilibrium dissociation constant (K_D_) of the scTCRs wt380 and s380. Dotted line indicates the K_D_ based on fitting the response to saturation. The K_D_ values are summarized in Table 3. (C) Biotinylated HLA-DQ2.5:CLIP2 was captured on neutravidin-coated sensor chips followed by injection of 5 **μ**M or 20 **μ**M of scTCRs. Representative sensograms of scTCR wt380 and scTCR s380 are shown (*n* = 1–3).

Next, we performed interaction studies using surface plasmon resonance (SPR) to determine binding affinity. Both scTCR wt380 and s380 bound their ligand HLA-DQ2.5:DQ2.5-glia-α1a, with apparent affinities of 30 μM and 7.5 μM, respectively (Fig 5A and 5B and Table 3) [21]. Thus, the affinity is in the 1–100 μM range typically observed for gluten-pMHC complexes [25, 29, 30], as well as for microbial-derived peptides [31, 32].

**Table 3:** SPR-derived kinetic and equilibrium constants.

TCR variant	K_D_ (eq.)	K_D_ (kd/ka)	ka (1/Ms)	kd (1/s)	Average K_D_^a^
scTCR wt380	27.3 ± 3.8x10^-6^ μM	32.5 μM	1.5x10^4^ ± 1.6x10^2^	0.5 ± 4.6x10^-3^	30 μM
scTCR s380	7.4 ± 7.0x10^-8^ μM	7.6 μM	9.5x10^4^ ± 1.3x10^3^	0.7 ± 9.8x10^-3^	7.5 μM

^a^Average K_D_ based on equilibrium and kinetic constants.

The binding response was higher for scTCR s380 than for wt380 at equal concentrations, indicating that wt380 contained a non-binding fraction. This is in line with the observations from the analytical SEC and DSF, further highlighting the necessity of stability engineering in order to obtain high quality SPR data. Neither of the scTCR preparations bound HLA-DQ2.5 with irrelevant peptide, CLIP2 (Fig 5C).

## Discussion and conclusions

Soluble TCRs are generally unstable and difficult to produce as functional molecules. In previous studies we have reported that over-expression of the chaperone FkpA has a major effect on soluble periplasmic expression of scTCRs, display levels on phage as well as selection performance of phage libraries [17, 20, 26]. In the current study, we utilize the same FkpA-assisted expression system and perform a side-by-side comparison of 4 different TCR formats; cFab, dsTCR, ct-dsTCR as well as wt and a stabilized version of scTCR. Furthermore, we investigate the influence of various *E*. *coli* host strains and induction of recombinant protein expression by IPTG.

Several *E*. *coli* strains are available for recombinant protein production, some of which are engineered for favorable traits. Here, we compared *E*. *coli* XL1-Blue and RosettaBlue^TM^. The latter strain expresses the pRARE plasmid that encodes tRNAs for codons that are rarely used by *E*. *coli*, thereby potentially enhancing the translation of eukaryotic genes containing such codons. Furthermore, culture conditions, such as induction, have been shown to influence protein production yields and subcellular localization. Enhanced expression due to IPTG induction of Lac promotor (LacPO)-regulated expression systems has previously been observed to result in decreased periplasmic protein yield [33], presumably due to clogging of the translocation system and overloading of the chaperones [34]. This may explain our observation that IPTG induction led to growth retardation in both XL1-Blue and RosettaBlue^TM^. In the absence of glucose and IPTG in the growth medium, leaky basal transcription occurs through the LacPO, resulting in reduced host stress due to lower translation and folding load. We have previously shown that these expression conditions are beneficial for soluble periplasmic scTCR expression [20], and here we extend the finding to include dsTCR, ct-dsTCR and cFab, none of which have previously been produced in a soluble periplasmic *E*. *coli* expression system. The combined effects of FkpA over-expression and reduced transcription, translation and folding load were essential for successful expression. Furthermore, the expression host RosettaBlue^TM^ generally gave a higher soluble yield than XL1-Blue. Thus, codon optimization or expression in a host engineered for eukaryotic protein expression is advised.

The TCR formats included in this study have different strengths and weaknesses as regards to downstream applications. Chimeric antibody variants, such as the cFab, can be highly useful as targeting unit e.g., as flow cytometric detection reagent in combination with commercially available secondary antibodies. This format, as well as the dsTCR variants, has the potential advantage of high stability, as the two chains are connected through a disulfide bond. However, as formation of disulfide bonds can only be efficiently achieved in the oxidizing environment of the *E*. *coli* periplasm, with the exception of using engineered *E*. *coli* strains [16], this is also an expression bottleneck. Furthermore, the two chains are expressed as separate polypeptide chains, which can result in differential expression and subsequent inefficient or erroneous chain pairing [35, 36]. Both the cFab and the dsTCRs were expressed as heterodimers. However, a substantial fraction was also detected as unpaired chains or aggregates. Thus, the folding machinery seems to be somewhat overloaded. It should be noted that the cFab format gave a higher yield of heterodimer than the dsTCR variants. The antibody molecule has evolved to be expressed as a soluble molecule, and fusion of unstable proteins to antibody domains has also previously enhances soluble expression, including of TCRs [14, 37]. Indeed, *E*. *coli* is capable of folding even the complex full-length antibodies in the periplasm at levels comparable to that reported here [24, 38]. Thus, further investigations into the cFab format could be warranted.

The scTCR variant has the advantage of being expressed as a single polypeptide chain and contains fewer disulfide bonds. However, this format is often prone to aggregation, and mutational assessment has pointed to three areas of critical importance for scTCR stability, namely amino acids in the Vα/Vβ domain interface, the Vβ HV4 region and surface residues normally shielded by the constant domains that become solvent exposed in the truncated form [22, 24, 38]. Lessons learned from these studies allow for rational protein stability engineering, which was carried out for the TCR380 used in this paper, which has a suboptimal Vα/Vβ domain interface [21]. Specifically, two mutations, L50P^TRAV^ and L103F^TRBV^, were introduced to form the triad shown to stabilize the Vα/Vβ interface, as L50^TRBV^ was already present. These mutations enable formation of a hydrophobic triad often found at the V_H_/V_L_ interface of the more stable antibody V domains [22]. In addition, we introduced a proline in position 2 of the linker (L2P^Linker^), as we have previously shown that this increases the thermostability of a scTCR [17]. In line with this observation, only the stabilized scTCR variant gave good yields of correctly folded monomeric protein. One would expect the stabilized molecule to have the highest thermal resistance [17, 39]. However, both DSF and intrinsic fluorescence spectroscopy used for thermal assessments showed that the scTCR wt380 exhibited a higher T_m_ than s380. Still, the Vα/Vβ domain interface was engineered to reduce aggregation propensity [40, 41], and the necessity for stability engineering in order to obtain high quality SPR data was demonstrated by the increased binding response observed for scTCR s380 compared to wt380. In SPR, the fraction of functional protein in the injected sample directly affects the estimated affinity values. Our data point to the value obtained for scTCR s380 to best reflect the true monomeric binding affinity, as this sample contains a larger functional fraction.

In a recent study, we employed this version of the stabilized scTCR s380, as well as a stabilized scTCR version of the HLA-DQ2.5:DQ2.5-glia-α2-reactive TCR364, scTCR s364, to evaluate the fine-specificities of the TCRs [21]. By use of these stabilized scTCRs we could carefully evaluate cross-reactivity to highly similar pMHC complexes.

In summary, we demonstrate that RosettaBlue^TM^ expression without IPTG induction results in the overall highest soluble periplasmic expression of all TCR formats tested. Under these conditions, we observed heterodimeric protein formation, and in particular formation of the cFab, and to a smaller extent the dsTCR. To our knowledge, this is the first time successful soluble periplasmic expression of heterodimeric TCRs is reported. Importantly, the periplasmic expression system used herein is compatible with high-throughput screening of TCR variants using 0.5 ml 96-well cultures. Periplasmic samples from these cultures can be analyzed by methods such as ELISA and western blotting. The stabilized version of the scTCR format was expressed in a functional yield that allowed for adequate TCR-pMHC interaction studies with SPR.

## Materials and methods

### Construction of TCR formats

The cloning of the V genes of the T-cell clone TCR380.E48 into the soluble expression vector pFKPEN [20] to create the **scTCR constructs** has been described before [21]. The pFKPEN vector is a derivative of the lacPO-based expression vector pHOG21 [33], but has been modified to constitutively express the periplasmic chaperone FkpA for efficient folding of heterologous proteins [20]. The **cFab construct** was generated by *NcoI*/*HindII* and *MluI*/*NotI* digestion of pFKPEN-VαβscTCRwt380 followed by sub-cloning of Vα and Vβ into the pFAB-Display phagemid vector on compatible RE sites [26]. The resulting cFab segment was then moved into pFKPEN as a *NcoI*/*SfiI* segment creating the pFABEFN-VαCH1-VβCκ-cFab380. The **ct-dsTCR construct** [13] was generated from mRNA previously isolated from TCC380.E48 using random hexamer primers (Promega) and SuperScriptII reverse transcriptase. Following RNaseH treatment and cDNA precipitation, entire TRA and TRB chains were retrieved using the gene-specific primers TRAV_NcoI_fw/TRA_Spe_rv and TRBV_Mlu_fw and TRB_Sfi_rv, respectively. The non-annealing tails of TRA_Spe_rv and TRB_Sfi_rv encode the antibody-derived C-terminal disulfide bridge. RE digested PCR products were subcloned into the RE digested pFABEFN vector generating the pFABEFN-VαCα-VβCβ-Ct-dsTCR380 vector. The C domain of the TRB chain contains a free cysteine which was removed by QuikChange mutagenesis using TRBC_C85.1A_fw and TRBC_C85.1A_rv primers to avoid aberrant disulphide bond formation. The ct-dsTCR vector generated above was used as a template to generate the **dsTCR construct** in a step-vise manner. First, non-native cysteines were introduced into TRAC and TRBC by QuikChange mutagenesis using the TRAC_T84C_fw/TRAC_T84C_rv and the TRBC_S79C_fw/TRBC_S79C_rv primer sets. Next, to abolish expression of the antibody-derived cysteine-containing tail of the ct-dsTCR, a stop codon was inserted immediately after TRAC using the TRAV_Nco_fw/dsTRA_Spe_rv primers. Finally, the cysteine-containing tail of TRBC was substituted for a read-through sequence leading into the C-terminal c-myc and His6-tags using the TRB_Mlu_fw/TRB_Sfi_v2_rv primers.

### Soluble periplasmic expression

Expression constructs were transformed into *E*. *coli* XL1-Blue and RosettaBlue^TM^ host strains. Small-scale expression was performed as follows; cells were inoculated from glycerol stocks into 5 ml 1x LB_TAG_ medium (1x LB medium supplemented with 100 μg/ml ampicillin, 20 μg/ml tetracycline and 0.1M glucose) or 1x LB_TACG_ medium (1x LB medium supplemented with 100 μg/ml ampicillin, 20 μg/ml tetracycline 30 μg/ml chloramphenicol and 0.1 M glucose) for XL1-Blue and RosettaBlue^TM^ strains, respectively. The cultures were grown at ON/37°C/220rpm, followed by re-inoculation to an OD_600nm_ of 0.025 and grown at 37°C/220rpm to an OD_600nm_ of 0.6–0.8. The cultures were spun down at 3220xg/30min and resuspended in 1x LB_TA_ or 1x LB_TAC_ medium either with or without 0.1 mM IPTG for induction, followed by incubation at ON/30°C/220rpm. Cells were pelleted by centrifugation at 3220xg/30min followed by cellular fractionation into medium, periplasmic and cytosolic fractions as described [20]. Large-scale expression (1 L cultures) was performed on the scTCR wt380, scTCR s380, cFab 380 and dsTCR 380 constructs in *E*. *coli* RosettaBlue^TM^ host strain without IPTG induction.

### Purification

Periplasmic fractions from large-scale expression were purified by IMAC on HisTrap HP (GE Healthcare) as described [20]. The eluted fractions were pooled and concentrated by Amicon Ultra (Millipore) followed by size exclusion chromatography (SEC) on a HiLoad 26/600 Superdex200 column (GE Healthcare). A final chromatography step of the samples on either ResourceQ or Superdex75 10/300 GL (both GE Healthcare) columns was performed when needed. The monomeric scTCR wt380 and scTCR s380 molecules or the heterodimeric dsTCR and cFab molecules were pooled and concentrated as before. SEC was performed using PBS with 300 mM NaCl and protein concentration was determined using the MW and extinction coefficient of each individual protein (Denovix DS-11^+^ Spectrophotometer). Analytical gelfiltration was performed using Superdex 200 Increase 3.2/300 column (GE Healthcare) run in PBS with 300 mM NaCl.

### SDS-PAGE and western blot analysis

For western blot analysis of medium, periplasmic and cytosolic fractions, cell cultures were measured by OD_600nm_ and normalized prior to fractionation. 15 μl of each fraction was mixed with loading buffer, heated 5 min at 95°C and run on a Criterion XT Precast 4–12% SDS-PAGE gel (Bio-Rad). Following semi-dry blotting, membranes were blocked with PBS with 4% (w/v) skim milk powder and detected using anti-Histidine tag-HRP antibody (1:10.000, AbD SeroTec, mouse monoclonal, clone AD1.1.10).

For analysis of purified TCRs, 2 μg each of sample was mixed BOLT^TM^ LDS sample buffer, heated 5 min at 95°C before separation on 12% NUPAGE BT gels in BOLT^TM^ MES SDS running buffer (reagents from Novex) along with Spectra prestained multicolor broad-range ladder (Thermo Scientific). Gels were stained with coomassie gel stain. Samples were reduced using DTT as indicated in the figures and figure legends.

### Expression of pMHC and purification

Recombinant HLA-DQ2.5 with the gluten-derived peptide DQ2.5-glia-α1a (QLQPFPQPELPY, underlined 9mer core sequence) or CLIP2 (MATPLLMQALPMGAL) coupled to the N-terminus of the HLA-DQ2.5 β-chain via a thrombin cleavable linker peptide was expressed in insect cells using a baculovirus expression vector system as previously described [42, 43]. Soluble, recombinant pMHC was affinity purified using the 2.12.E11 antibody specific for HLA-DQ2 (mouse monoclonal, clone 2.12.E11 [44]). After site-specific biotinylation using BirA (Avidity) as previously described [42], pMHCs were purified using Superdex200 GL10/300 run in PBS.

### Differential scanning fluorimetry (DSF)

Protein stability of the purified monomeric scTCRs was measured by DSF using a LightCycler 480 II RT-PCR machine (Roche). SYPRO Orange (Sigma) was used at a 1:1000 dilution and protein concentration at 0.1 mg/ml in a volume of 25 μl. Samples were run in triplicates in 96-well Lightcycler 480 Multiwell Plate. The RT-PCR machine was programmed to ramp the temperature from 20°C to 90°C after a stabilization period for 10 min at 20°C. Data were collected every 0.5°C using the 465 nm excitation and 580 nm emission filters. Data transformation and analysis was performed using the DSF Analysis protocol essentially as described [45].

### Fluorescence spectroscopy

The thermostabilities of scTCRs were determined from measurements of intrinsic tryptophan fluorescence during exposure to a temperature gradient from 20°C to 90°C at 1°C/min. Measurements were done in a Jasco-8500 spectrofluorometer equipped with a Peltier temperature control unit using 3 μM proteins in PBS with 300 mM NaCl. 300 μL sample was placed in a 5x5 mm quartz cuvette (HellmaAnalytics). The sample was stirred with a magnetic bead at 150 rpm during the entire measurement. Fluorescence was measured at 1°C intervals during the gradient. The sample was excited at 295 nm and emission measured at 350 nm with medium sensitivity of the photo multiplier (PMT voltage 385V). A marked increase in fluorescence was interpreted as a transition from native to denatured protein. Background was subtracted using the spectra analysis software (Jasco). The relative fluorescence intensity was plotted versus temperature for determination and comparison of T_m_ using Graphpad Prism 7.

### Surface plasmon resonance (SPR)

SPR was performed using a Biacore T200 instrument (GE Healthcare). Neutravidin (10 μg/ml in acetate buffer pH 4.5) was immobilized on a CM3 Series S sensor chip by amine coupling to 1000 resonance units (RU), followed by capture of approximately 100 RU of HLA-DQ2.5:DQ2.5-glia-α1a or HLA-DQ2.5:CLIP2. A 2 fold dilution series ranging from 40 μM to 2.5 μM of soluble scTCR wt380 or scTCR s380 were injected over the HLA-DQ2.5:DQ2.5-glia-α1a-coated surface, while 20, 5 and 0 μM were injected over the HLA-DQ2.5:CLIP2-coated surface. All experiments were run at a flow rate of 30 μl/min at 25^○^C using single cycle kinetics method (data collection rate 10 Hz). In all experiments, data were zero-adjusted and the Neutravidin reference flow cell value subtracted before analysis using the T200 Evaluation software. A 1:1 Langmuir binding model was used for determination of K_D_ (RI set to constant).

## Supporting information

S1 Fig Amino acid sequences of the TCR constructs. The amino acid sequence of (A) scTCR wt380, (B) scTCR s380, (C) dsTCR 380, (D) ct-dsTCR 380 and (E) cFab 380. In all variants the leader sequence is highlighted in light grey, variable domains in white, linker in teal and c-Myc and His6 tags in violet. The introduced stabilizing mutations of scTCR s380 (B) is highlighted in red, while stabilizing amino acids already present in the sequence are highlighted in dark red. The TCR constant domains of the dsTCR (C) and ct-dsTCR (D) are highlighted in dark grey and the cysteine residues forming the introduced disulfide bridge are highlighted in pink. The antibody constant of the cFab (D) domains are highlighted in turquoise.
(TIF)

S2 Fig Expression characteristics of the recombinant TCR formats. Representative western blot showing the expression profile of the TCR formats. *E*. *coli* RosettaBlue^TM^ expression cultures were normalized and fractionated into; M, medium; P, periplasmic; C, cytosolic fractions before analysis by western blots (*n* = 3–4). All samples were detected with anti-His-HRP antibody. Expression of scFv anti-phOx was included as positive control.
(TIF)

S3 Fig Original uncropped western blots. Fig 2A are composed of the following three (A, B, C) uncropped original western blots.
(TIF)

S1 Table Oligonucleotides. (PDF)
