# Supplementary figures and images for: Soluble T-cell receptor design influences functional yield in an E. coli chaperone-assisted expression system
Source: PLoS One. 2018 Apr 12;13(4):e0195868. doi: 10.1371/journal.pone.0195868 (PMC5897000; doi:10.1371/journal.pone.0195868)

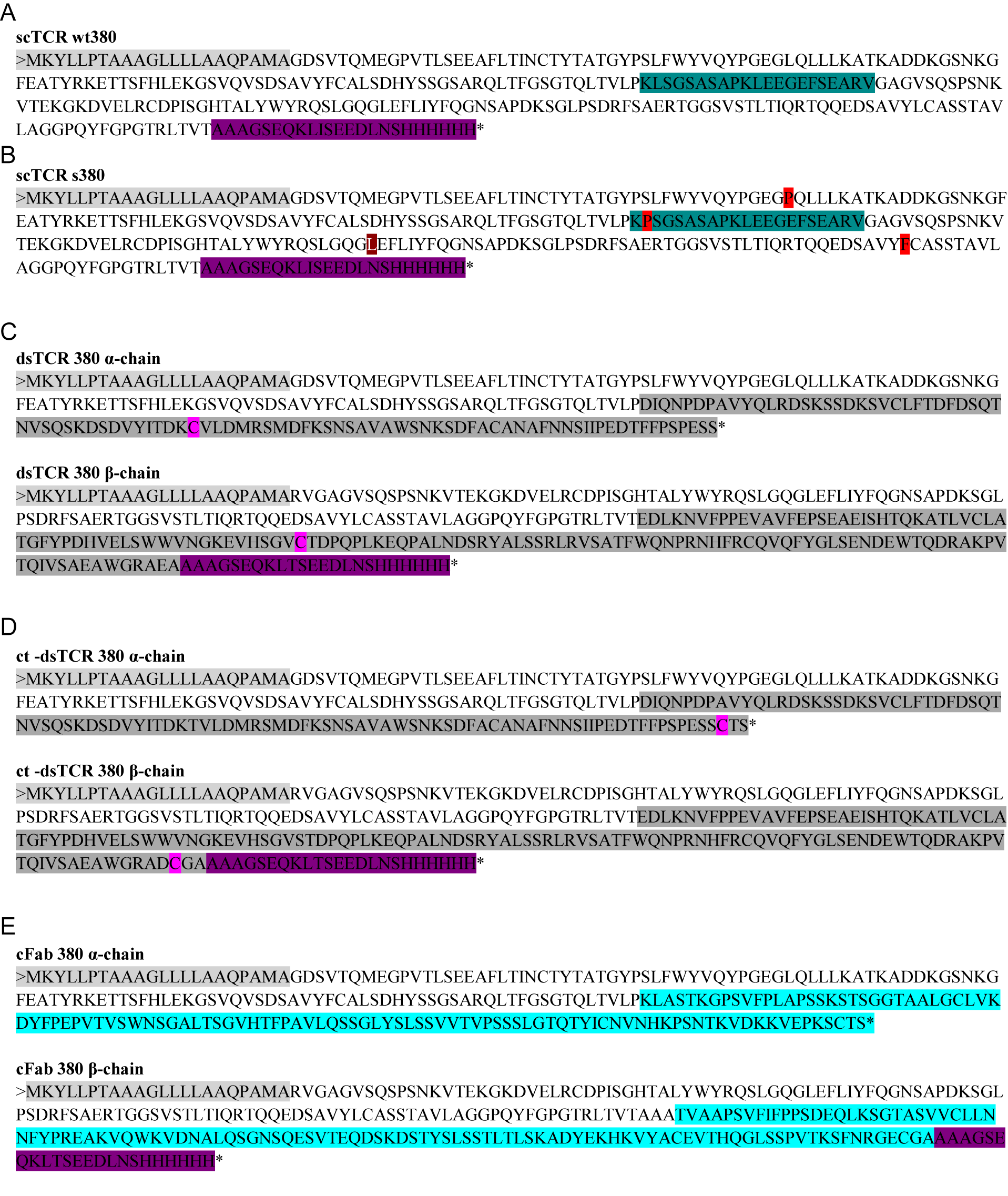

Supplement: S1 Fig — The amino acid sequence of (A) scTCR wt380, (B) scTCR s380, (C) dsTCR 380, (D) ct-dsTCR 380 and (E) cFab 380. In all variants the leader sequence is highlighted in light grey, variable domains in white, linker in teal and c-Myc and His6 tags in violet. The introduced stabilizing mutations of scTCR s380 (B) is highlighted in red, while stabilizing amino acids already present in the sequence are highlighted in dark red. The TCR constant domains of the dsTCR (C) and ct-dsTCR (D) are highlighted in dark grey and the cysteine residues forming the introduced disulfide bridge are highlighted in pink. The antibody constant of the cFab (D) domains are highlighted in turquoise. (TIF) [file pone.0195868.s001.tif]

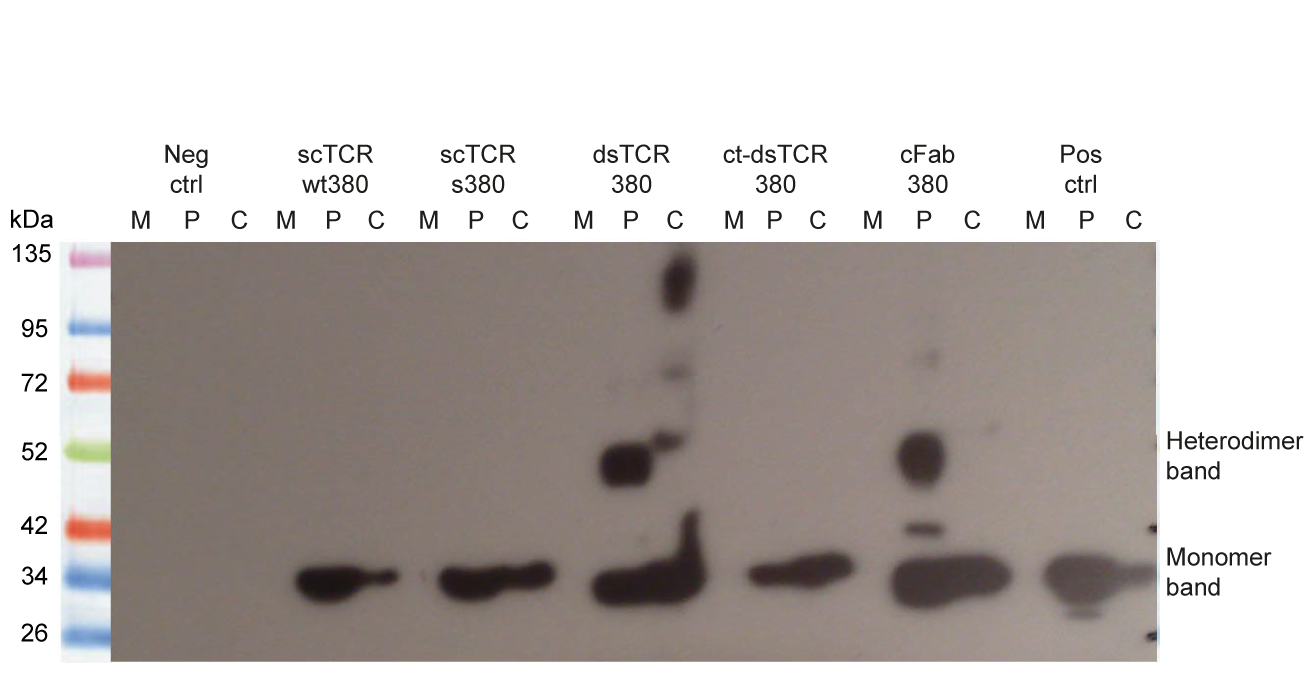

Supplement: S2 Fig — Representative western blot showing the expression profile of the TCR formats. E. coli RosettaBlueTM expression cultures were normalized and fractionated into; M, medium; P, periplasmic; C, cytosolic fractions before analysis by western blots (n = 3–4). All samples were detected with anti-His-HRP antibody. Expression of scFv anti-phOx was included as positive control. (TIF) [file pone.0195868.s002.tif]

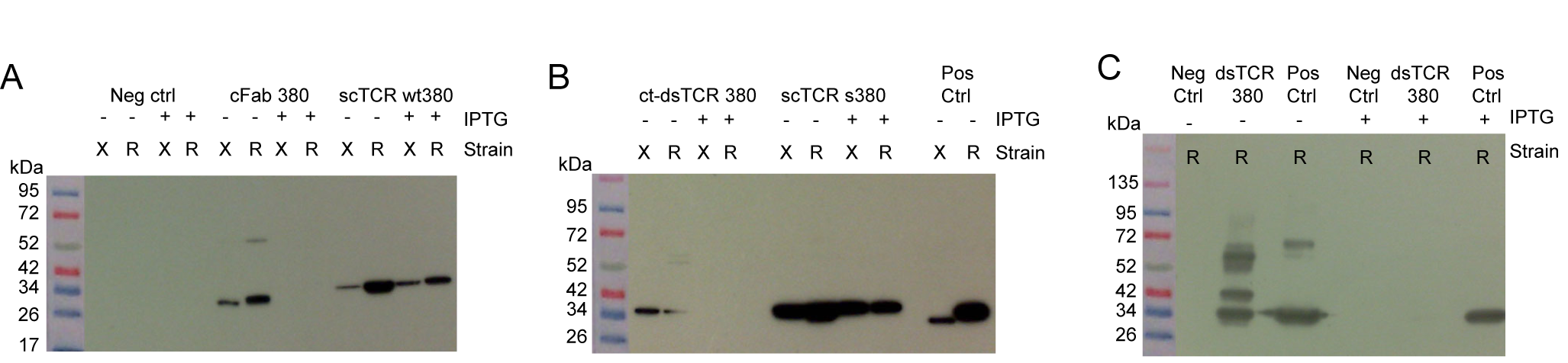

Supplement: S3 Fig — Fig 2A are composed of the following three (A, B, C) uncropped original western blots. (TIF) [file pone.0195868.s003.tif]
