# Supplementary material for: Soluble T-cell receptor design influences functional yield in an E. coli chaperone-assisted expression system
Source: PLoS One. 2018 Apr 12;13(4):e0195868. doi: 10.1371/journal.pone.0195868 (PMC5897000; doi:10.1371/journal.pone.0195868)
Supplement: S1 Table — (PDF) [file pone.0195868.s004.pdf]

S1 Table. Oligonucleotides

TRAC_rv	5'-AGTCAGATTTGTTGCTCCAGGCC-3'
TRBC_rv	5'-TTCACCCACCAGCTCAGCTCC-3'
poly-G-NotI_fw	5'-ATATGCGGCCGCGGGGGGGGGGGGGGGG-3'
TRAC_MluI_rv	5'-ATACGCGTTCTCTCAGCTGGTACACGG-3'
TRBC_MluI_rv	5'-ATACGCGTAGATCTCTGCTTCTGATGGC-3'
TRAV_NcoI_fw	5'- ATCCATGGCCGGAGATTCAGTGACCCAGATGG-3'
TRAV_HindII_rv	5'- ATATATAAGCTTAGGTAAAACAGTCAATTGTGTCCC-3'
TRBV_MluI_fw	5'- ATATACGCGTAGGAGCTGGAGTCTCCCAG-3'
TRBV_NotI_rv	5'- ATATGCGGCCGCTGTGACCGTGAGCCTGG-3'
TRAV_L50P_fw	5'-AATATCCTGGAGAAGGTCCGCAGCTCCTCCTGAAAGCC-3'
TRAV_L50P_rv	5'-GGCTTTCAGGAGGAGCTGCGGACCTTCTCCAGGATATT-3'
Linker_L2P_fw	5'-TGACTGTTTTACCTAAGCCGTCAGGGAGTGCATCCGCC-3'
Linker_L2P_rv	5'-GGCGGATGCACTCCCTGACGGCTTAGGTAAAACAGTCA-3'
TRBV_L103F_fw	5'-CAGGAGGACTCGGCCGTGTATTTTTGTGCCAGCAG-3'
TRBV_L103F_rv	5'-CTGCTGGCACAAAAATACACGGCCGAGTCCTCCTG-3'
TRA_Spe_rv	5'-ATACTAGTGCAGGAACTTTCTGGGCTGGGG-3'
TRB_Sfi_rv	5'-ATATGGCCGCAGCGGCCCCACAGTCTGCTCTACCCCAGGCC-3'
dsTRA_spe_rv	5'-ATACTAGTTCATTAGGAACTTTCTGGGCTGG-3'
TRB_sfi_v2	5'-ATATGGCCGCAGCGGCCTCTGCTCTACCCCAGGCC-3'
TRBC_C85.1A_fw	5'PHO-TCAATGACTCCAGATACGCGCTGAGCAGCCGCCTGAG-3'
TRBC_C85.1A_rv	5'PHO-CTCAGGCGGCTGCTCAGCGCGTATCTGGAGTCATTGA-3'
TRAC_T84C_fw	5'-TCTGATGTGTATATCACAGACAAATGCGTGCTAGACATGAGGTCTATGG-3'
TRAC_T84C_rv	5'-CCATAGACCTCATGTCTAGCACGCATTTGTCTGTGATATACACATCAGA-3'
TRBC_S79C_fw	5'-CACAGTGGGGTCTGCACAGACCCGC-3'
TRBC_S79C_rv	5'-GCGGGTCTGTGCAGACCCCACTGTG-3'

All primers were purchased from Eurofins
